# A fragrant neighborhood: volatile mediated bacterial interactions in soil

**DOI:** 10.3389/fmicb.2015.01212

**Published:** 2015-11-03

**Authors:** Kristin Schulz-Bohm, Hans Zweers, Wietse de Boer, Paolina Garbeva

**Affiliations:** ^1^Department of Microbial Ecology, Netherlands Institute of Ecology (NIOO-KNAW)Wageningen, Netherlands; ^2^Department of Soil Quality, Wageningen UniversityWageningen, Netherlands

**Keywords:** volatiles, inter-specific interactions, rhizosphere, synthetic microbial communities, low-abundant bacteria, soil microcosms

## Abstract

There is increasing evidence that volatile organic compounds (VOCs) play essential roles in communication and competition between soil microorganisms. Here we assessed volatile-mediated interactions of a synthetic microbial community in a model system that mimics the natural conditions in the heterogeneous soil environment along the rhizosphere. Phylogenetic different soil bacterial isolates (*Burkholderia sp., Dyella sp., Janthinobacterium sp., Pseudomonas sp.*, and *Paenibacillus sp.*) were inoculated as mixtures or monoculture in organic-poor, sandy soil containing artificial root exudates (ARE) and the volatile profile and growth were analyzed. Additionally, a two-compartment system was used to test if volatiles produced by inter-specific interactions in the rhizosphere can stimulate the activity of starving bacteria in the surrounding, nutrient-depleted soil. The obtained results revealed that both microbial interactions and shifts in microbial community composition had a strong effect on the volatile emission. Interestingly, the presence of a slow-growing, low abundant *Paenibacillus* strain significantly affected the volatile production by the other abundant members of the bacterial community as well as the growth of the interacting strains. Furthermore, volatiles released by mixtures of root-exudates consuming bacteria stimulated the activity and growth of starved bacteria. Besides growth stimulation, also an inhibition in growth was observed for starving bacteria exposed to microbial volatiles. The current work suggests that volatiles produced during microbial interactions in the rhizosphere have a significant long distance effect on microorganisms in the surrounding, nutrient-depleted soil.

## Introduction

Microorganisms produce a great variety of secondary metabolites including antibiotics, toxins, pigments, and others. Interestingly, small molecular mass metabolites such as volatile organic compounds (VOCs) were for a long time overlooked. Research of the last decades demonstrated that bacteria produce a large set of VOCs (Kai et al., 2009; Insam and Seewald, 2010; Effmert et al., 2012; Bitas et al., 2013; Peñuelas et al., 2014). However, the knowledge about the biological and ecological function of those volatiles is still limited. Similar to well-studied plant VOCs, it can be assumed that VOCs released by bacteria perform diverse and crucial functions (Bitas et al., 2013). Recent studies revealed that bacterial volatiles can inhibit the growth of fungi or bacteria (Wrigley, 2004; Kai et al., 2007; Vespermann et al., 2007; Zou et al., 2007; Weise et al., 2012; Garbeva et al., 2014b) and in some cases they can even function as growth-promoting agent (Wheatley, 2002; Horii and Ishii, 2006; Garbeva et al., 2014a). Additionally, volatiles emitted by bacteria can influence the metabolism of other surrounding bacteria (Kai et al., 2009; Garbeva et al., 2014a). Most studies, however, are performed *in vitro* on semi-solid media using nutrient rich conditions and may not represent the natural conditions in the microbial environment. Culture conditions including nutrient availability (Kai et al., 2009; Insam and Seewald, 2010) and the type of incubation medium substantially affect the spectrum of released VOCs (Weise et al., 2012). Thus, bacteria produce a different set of volatiles when incubated in soil as compared to incubations on agar plates (Garbeva et al., 2014b).

Soil is a complex, nutrient-poor and highly heterogeneous environment consisting of both water- and air-filled pores (Young et al., 2008). Due to their physical properties such as low molecular weight (< 300 D), lipophilicity, high vapor pressure and low boiling points (Effmert et al., 2012; Bitas et al., 2013; Lemfack et al., 2014), VOCs can diffuse through water- and gas-filled pores in soil and rhizosphere environments. Therefore, VOCs can act over a wider range of scale and may play essential roles in the communication and competition between physically separated microorganisms in soil (Kai et al., 2009; Effmert et al., 2012; Garbeva et al., 2014a). Soil microbes occur generally in multi-species communities. However, our current knowledge on the ecological role of microbial volatiles is based mostly on studies focusing on volatiles released by (non-interacting) monocultures. During inter-specific interactions, the production of various secondary metabolites can be triggered (e.g., Garbeva and De Boer, 2009; Traxler et al., 2013) and these secondary metabolites can act differently as compared to metabolites released by species in monoculture (Garbeva et al., 2014a; Tyc et al., 2014).

In the present study, we aim to reveal new insights into the ecological role of VOCs in microbial interactions by assessing multi-species interactions of a synthetic microbial community in a soil model system that reflects the natural conditions in the heterogeneous soil environment along the rhizosphere. The main research questions to address were: (1) what is the role of microbial interactions and shifts in microbial community composition on volatile emission in the rhizosphere and (2) can volatiles produced during bacterial interactions in the rhizosphere stimulate the activity of starving soil microbes in the surrounding environment?

Overall, our results demonstrated that both microbial interactions and shifts in microbial community composition had a significant effect on the volatile emission and that the presence of slow growing, non-abundant bacterial species influenced the volatile production of the bacterial community. Furthermore, our results revealed that volatiles released by microbial interactions in the rhizosphere have a long distance effect on the surrounding non-active microbial community in the nutrient-depleted soil.

## Materials and methods

### Bacterial model strains and growth media

Bacterial strains (Table 1) were previously isolated from the rhizosphere of sand sedge (*Carex arenaria*) in different sandy dune soil sites (De Ridder-Duine et al., 2005) and characterized by 16S rRNA gene sequencing (Tyc et al., 2014). All bacterial isolates were pre-cultured from frozen glycerol stocks on 0.1 strength tryptone soya broth (TSB) agar (De Boer et al., 2007). Overnight cultures in 0.1 TSB medium were prepared prior to each microcosm experiment.

**Table 1:** **Bacterial strains used in this study**.

**Bacterial strain (Accesion number)**	**Origin**	**Phylum *(family)***
*Achromobacter spp.* 58-38 (KC888968)	River dune near Bergharen, Gelderland (De Boer et al., 2015)	Beta-Proteobacteria *(Alcaligenaceae)*
*Bosea sp.* AD132 (KJ685339)	Coastal outer dunes of Midsland, Terschelling (De Ridder-Duine et al., 2005)	Alpha-Proteobacteria *(Bradyrhizobiaceae)*
*Burkholderia sp.* AD024 (KJ685239)	Dune grassland near Ouddorp, Zeeland (De Ridder-Duine et al., 2005)	Beta-Proteobacteria *(Burkholderiaceae)*
*Dyella sp.* AD056 (KJ685269)	Drift sand near Loon op Zand, Brabant (De Ridder-Duine et al., 2005)	Gamma-Proteobacteria (*Xanthomonadaceae*)
*Janthinobacterium sp.* AD080 (KJ685292)	Coastal outer dunes of Midsland, Terschelling (De Ridder-Duine et al., 2005)	Beta-Proteobacteria *(Oxalobacteraceae)*
*Microbacterium sp.* AD141 (KJ685346)	Costal outer dunes of EastTerschelling (De Ridder-Duine et al., 2005)	Actinobacteria (*Microbacteriaceae*)
*Paenibacillus sp.* AD087 (KJ685299)	Pine plantation near Loon op Zand, Brabant (De Ridder-Duine et al., 2005)	Firmicutes (Paenibacillaceae)
*Pedobacter sp.* V48 (NZ_AWRU00000000)	Coastel dune site Terschelling (De Boer et al., 1998)	Bacteroidetes *(Sphingobacteria)*
*Pseudomonas sp.* AD021 (DQ778036)	Coastel inner dunes of Midsland, Terschelling (De Ridder-Duine et al., 2005)	Gamma-Proteobacteria *(Pseudomonadaceae)*
*Rhizobium sp.* 45-29 (KC888976)	River dune near Bergharen, Gelderland (De Boer et al., 2015)	Alpha-Proteobacteria *(Rhizobiaceae)*

All strains are from organic poor, sandy dune soils in the Netherlands.

### Rhizospheric soil microcosms

Sandy soil of low carbon-content (Figure S1) and low amount of mineral nitrogen (0.2 mg/kg nitrite and nitrate) and phosphate (1.1 mg/kg) was collected from an old river dune site near the village Bergharen (51°10′N, 05°40′E) in the Netherlands. The soil was dried, sieved (ø 2 mm), and gamma-sterilized by Synergy Health Ede B.V. (Netherlands). Before each microcosm experiment, the gamma-sterilized soil was acclimatized for 5 days under sterile conditions. Artificial Root Exudates (ARE) stock solution (7.5 mg carbon per ml, C/N 20.6) containing 18.4 mM glucose, 18.4 mM fructose, 9.2 mM sucrose, 9.2 mM citric acid, 4.6 mM fumaric acid, 20.5 mM lactic acid, 6.9 mM malic acid, 13.8 mM succinic acid, 2.0 mM cysteine, 6.1 mM L-alanine, 2.0 mM L-argenine, 3.6 mM L-glutamic acid, 6.1 mM L-serine, and 10 mM KH_2_PO_4_ was freshly prepared or stored at −20°C. The composition of the ARE stock solution was based on previously published ARE-mixes (Griffiths et al., 1999; Baudoin et al., 2003; Garbeva et al., 2014b) and adapted to root-exudate composition found for the sedge *Schoenus unispiculatus* (Shane et al., 2006) belonging to the same family Cyperaceae as *C. arenaria.* Liquid inoculums consisting of bacteria (10^8^ cells ml^−1^ per strain), ARE stock solution (pH 6.5), and phosphate buffer (10 mM KH_2_PO_4_, pH 6.5) were prepared. The inoculums were thoroughly mixed with the soil to establish rhizospheric soil microcosms containing 136 μg carbon per g soil and 1 × 10^5^ CFU per g soil for each strain. The initial carbon concentration in the soil microcosms was adjusted to estimated daily carbon inputs by roots of 100–1500 μg carbon per g rhizosphere soil (Trofymow et al., 1987; Cheng et al., 1996). Control microcosms consisted of sterile soil, phosphate buffer and ARE stock solution without bacterial inoculation. The moisture content was 5.7% (w/w). Microcosms were incubated at 20°C.

### Bioassay for the selection of bacterial strains

Three soil microcosms for each bacterial strain were set up in 25 ml gas tight glass bottles as described above. The head space in the bottles was filled with atmospheric gas. All microcosms were incubated for 7 days at 20°C and the bottles were flushed with fresh air every 2 days. Gas samples were taken before and after flushing with fresh air and the CO_2_ production was measured with an Ultra GC gas chromatograph (Interscience, The Netherlands) equipped with a flame ionization detector (FID) and a Rt-QBond (30 m, 0.32 mm, ID; Restek, USA) capillary column. Carrier gas was helium at a flow rate of 5 ml min^−1^. Injector, oven and detector temperatures were 150, 50, and 350°C, respectively. CO_2_ concentrations in the headspace were calculated based on external gas standards (Westfalen AG, Germany) and the ideal gas law.

Soil samples were regularly taken to monitor the bacterial growth. For this 1 g of soil was mixed with 9 ml phosphate buffer (pH 6.5) in 20 ml Greiner tubes. The tubes were shaken on a rotary shaker at 180 rpm for 30 min at 20°C and serial dilutions were plated on 0.1 TSB agar. All plates were incubated at 20°C and colony forming units (CFU) were counted after 3 days.

### Collection of bacterial volatiles and monitoring of bacterial growth in rhizospheric soil microcosms

For the collection of bacterial VOCs, rhizospheric soil microcosms were established as indicated above in glass Petri-dishes with an exit at the top to which a steel trap filled with 150 mg Tenax TA and 150 mg Carbopack B (Markes International Ltd., Llantrisant, UK) could be fixed. In the rhizospheric soil microcosms, the bacterial strains *Burkholderia* sp. AD024, *Dyella* sp. AD056, *Janthinobacterium* sp. AD080, *Pseudomonas* sp. AD021, and *Paenibacillus* sp. AD087 were either incubated as monocultures or as mixtures of four (without *Paenibacillus sp*. AD087) or five strains. The growth of the strains was monitored by quantification of bacterial 16S rRNA genes from extracted DNA of soil samples (three per microcosm) taken at the beginning of the experiment and after 96 h incubation. Soil samples were stored at −80°C until nucleic acid extraction (see below). VOCs produced by the monocultures, bacterial mixtures and control (see above) were trapped for 24 h after 3 days of incubation. Traps were removed, capped and stored at 4°C until analysis.

### VOCs analysis

VOCs were desorbed from the traps using an automated thermodesorption unit (model UnityTD-100, Markes International Ltd., UK) at 210°C for 12 min (He flow 50 ml/min). The desorbed VOCs were subsequently collected on a cold trap at −10°C and introduced into the GC-QTOF (model Agilent 7890B GC and the Agilent 7200A QTOF, USA) by heating the cold trap for 3 min to 280°C. Split ratio was set to 1:201, and the column used was a 30 × 0.25 mm ID RXI-5MS with as film thickness of 0.25 μm (Restek 13424-6850, Bellefonte, PA, USA). The temperature program was as follows: 2 min at 39°C, 3,5°C/min to 95°C, 6°C/min to 165°C, 15°C/min to 250°C and finally 40°C/min to 300°C which was hold for 20 min. VOCs were detected by the MS operating at 70 eV in EI mode. Mass spectra were acquired in full scan mode (30–400 AMU, 4 spectras/s). GC/MS data were collected and converted to mzData file using the Chemstation B.06.00 (Agilent Technologies, USA). Data were further processed with MZmine 2.14.2 (Pluskal et al., 2010) with the tools mass detection (centroid mode, noise level = 1000), chromatogram builder (Min time span = 0.04 min, Min height = 1.8E04–2.5E04, m/z tolerance of 1 m/z or 5 ppm), and chromatogram deconvolution (local minimum search, chromatographic threshold = 40%, Min in RT range = 0.1 min, Min relative height = 2.0%, Min absolute height = 1.5E04, Min ratio of peak top/edge = 2, peak duration = 0.0–0.7 min). Detected and deconvoluted peaks were identified by their mass spectra using NIST MS Search and NIST 2014 (National Institute of Standards and Technology, USA,http://www.nist.gov) and aligned by RANSAC aligner (mz tolerance = 1 m/z or 5 ppm, RT tolerance = 0.1, RT tolerance after correction = 0.05, RANSAC iteration = 10000, Min number of points = 60%, threshold value = 0.1). Processed data were exported for further statistical analysis (see below). The identification of detected compounds was further evaluated by using the software AMDIS 2.72. The retention indexes were calculated for each compound and compared with those found in NIST 2014 and in house databases. Detected compounds were named as identified compounds if the Match and Reverse-Match factor of the listed compound in the NIST and in house NIOO-library was higher than 800, the spectra of the detected compound matches the one of the listed compound, and the difference between the retention index calculated for the detected compound and of the listed compound (for a semi-standard non-polar column) was not bigger than four. The Match and Reverse match factor refer to the similarity of the mass spectrum of the sample with the library spectrum of the Hit (Watson and Sparkman, 2013). Some identified compounds were also verified by co-injection of pure compounds (Table 2).

**Table 2:** **Volatile organic compounds produced by a bacterial mixture of five strains (*Burkholderia sp.* AD024, *Dyella sp.* AD056, *Janthinobacterium sp.* AD080, *Pseudomonas sp.* AD021, and *Paenibacillus sp.* AD087), referred to as 5-Mix, or four strains excluding *Paenibacillus sp.* AD087, referred to as 4-Mix**.

**Compound**	**Class**	**RI**
**COMPOUNDS DETECTED FOR 4-MIX AND 5-MIX**		
2-Pentanone^a^^,^^c^	Ketone	683
3-Pentanone^a^^,^^c^	Ketone	695
Unknown^c^	n.s.	709
^*^Dimethyl disulfide^b^^,^^c^^,^^e^	Organosulfur	744
2-Octanol^a^^,^^c^	Alcohol	996
L-Fenchone^a^^,^^c^	Monoterpene, Ketone	1088
Unknown^d^	n.s.	1091
Camphor	Terpenoid	1144
Unknown	n.s.	1316
2,6-Bis(1,1-dimethylethyl)-2,5-Cyclohexadiene-1,4-dione (DBQ)	Aromate/Phenol	1461
Butylated Hydroxytoluene^a^^,^^b^^,^^c^^,^^d^^,^^e^	Aromate	1501
Unknown^c^	n.s.	1515
2,2,4-Trimethyl-1,3-pentanediol diisobutyrate^a^^,^^e^	Ester	1588
Unknown^b^^,^^c^^,^^d^	n.s.	1718
1,2-Benzenedicarboxylic acid, bis(2-methylpropyl) ester^a^^,^^b^	Ester	1874
**COMPOUNDS DETECTED FOR 4-MIX**		
Methoxy-acetaldehyde^b^^,^^d^	Aldehyde	559
Cyclopentene^a^^,^^b^	Alkene	570
tert-Butanol^a^^,^^b^^,^^c^^,^^e^	Alcohol	575
Acetic acid	Organic acid	593
2-methyl-2-propen-1-ol^a^^,^^b^^,^^c^	Alcohol	605
Ethyl benzene	Aromate	857
3-Heptanol^b^^,^^c^^,^^e^	Alcohol	896
n-Hexadecanoic acid	Organic acid	1964
**COMPOUNDS DETECTED FOR 5-MIX**		
Sulfur dioxide^a^	Organosulfur	548
1,3,5-Trifluorobenzene^a^^,^^b^	Aromate	624
Propanoic acid, 2,2-dimethyl-, methyl ester^b^	Ester	720
2-Hexanone^c^^,^^d^	Ketone	790
2-Heptanone^c^	Ketone	888
Unknown	n.s.	1020
Methyl 2-ethylhexanoate	Ester	1039
Endo-borneol^a^^,^^b^^,^^d^	Terpene	1167
^*^2,5-Bis(1-methylethy)-pyrazine	Aromate	1185
Unknown	n.s.	1200
Unknown	n.s.	1231
Unknown^a^^,^^c^	n.s.	1244
Unknown	n.s.	1283
Unknown	n.s.	1343
Unknown^a^^,^^c^	n.s.	1690
^*^Docosane^b^	Alkene	2200

Bacteria were incubated for 4 days in soil supplied by ARE.

a Volatiles also detected for monocultures of Burkholderia sp. AD024;

b Volatiles also detected for monocultures of Dyella sp. AD056;

c Volatiles also detected for monocultures of Janthinobacterium sp. AD080;

d Volatiles also detected for monocultures of Paenibacillus sp. AD087;

e Volatiles also detected for monocultures of Pseudomonas sp. AD021;

RI Linear retention Index of a 30 × 0.25 mm ID RXI-5MS column;

n.s., not specified;

* Verified by co-injection of pure compound

### Bioassay to test the effect of microbial VOCs on nutrient-limited bacteria in soil

Soil microcosms containing the bacterial strains *Burkholderia* sp. AD024, *Dyella* sp. AD056, *Janthinobacterium* sp. AD080, *Pseudomonas* sp. AD021, and *Paenibacillus* sp. AD087 were set-up in two-compartment Petri-dishes (**Figure 2** and Figure S3). Per Petri-dish, one nutrient-limited compartment was established containing 20 g gamma-sterilized soil mixed with 1 × 10^5^ CFU per g soil of each strain and phosphate buffer (10 mM KH_2_PO_4_, pH 6.5). The second compartment was either filled with a mix of 20 g soil, phosphate buffer and ARE stock solution (138 μg carbon per g soil), referred to as control, or a mix of 20 g soil, the five bacterial strains (1 × 10^5^ CFU per g soil of each strain), phosphate buffer and ARE stock solution, referred to as Treatment 1 or Treatment 2 (**Figure 3A** and Figure S3A). In case of Treatment 2, bacteria in the nutrient-limited compartment were pre-incubated for 2 days before the bacteria-ARE-soil-mix was added to the second compartment. It can be assumed that during the pre-incubation available carbon sources in the soil should be fully consumed so that bacteria were strongly nutrient limited before they were exposed to microbial volatiles of the second compartment. The composition of the ARE containing compartment is equivalent to the rhizospheric microcosms described above. The moisture content of all compartments was 5.8% (w/w). All samples were set-up in triplicates and incubated at 20°C for 6 days. To monitor bacterial activity and potential growth, about 1.5 g soil were taken two times per compartment at the start of the experiment and after 2, 4, and 6 days of incubation. For nucleic acid extraction, 0.5 g of the collected soil was immediately treated with a salmon sperm DNA solution and freeze-dried at −80°C. Leftover soil collected was stored as backup at −80°C.

### Nucleic acid extraction

A solution (~pH 8) of 10 mg per ml low molecular weight salmon sperm DNA (Sigma-Aldrich, The Netherlands) was prepared with DNase- and RNase-free water (Qiagen, The Netherlands). According to Paulin et al. (2013), 0.5 g of soil per sample was mixed with 0.5 ml of salmon sperm DNA solution in Lysing Matrix E tubes (MP Biomedicals, The Netherlands) and freeze-dried overnight at −80°C. DNA and RNA were co-extracted using the modified protocol described by Griffiths et al. (2000). Briefly, 500 μl of cetyl-trimethyl ammonium bromide (CTAB) buffer (10% CTAB in 0.7 M NaCl mixed with 1 volume 240 mM phosphate buffer pH 8.0), 50 μl of 2% N-lauroyl sarcosine, 50 μl of 2% sodium dodecyl sulfate, and 400 μl of phenol-chloroform-isoamyl alcohol (25:24:1, Sigma-Aldrich, The Netherlands) were added to the freeze-dried soil. A subsequent bead beating lysis for 30 s at 5.5 ms^−1^ was performed three-times followed by 7 min centrifugation at 14,000 rpm and 4°C. The resulting aqueous supernatant was mixed with 1 volume (vol.) of chloroform-isoamyl alcohol (24:1, Sigma-Aldrich, The Netherlands) and centrifuged for 5 min at 14,000 rpm and 4°C. After a second phenol extraction with 1 vol. chloroform-isoamyl alcohol the aqueous supernatant was thoroughly mixed with 2 vol. of 20% polyethylene glycol 6000 (AppliChem, Germany) in 1.6 M NaCl, 0.1 vol of 100 mM MgCl_2_, and 1.5 μl glycogen (20 mg ml^−1^; Thermo Fisher Scientific, The Netherlands). Nucleic acids were precipitated for 2 h at 4°C, followed by 40 min centrifugation at 14,000 rpm and 4°C. The pellet was washed with 70% ethanol, air-dried and eluted in 37–65 μl DNase- and RNase-free water. Nucleic acid extracts were stored at −80°C or subsequently used for quantification of bacterial 16S rRNA genes (see below).

### DNase treatment and reverse transcription

Nucleic acid extracts were treated according to the manufacturer's protocol wi th 1–2 U DNase I (Thermo Fisher Scientific, The Netherlands) and incubated for 45 min at 37°C. RNA was purified by an overnight precipitation at −20°C with 0.1 vol. ammonium acetate (2.5 mM, Sigma-Aldrich), 2.5 vol. 100% ethanol, and 1.3 μl glycogen, followed by centrifugation for 40 min at 13,000 rpm and 4°C. The RNA pellet was washed twice with 70% ethanol and eluted in 27 μl DNase- and RNase-free water. RNA was stored at −80°C or subsequently used for reverse transcription with SuperScript® III First-Strand Synthesis System (Invitrogen, The Netherlands). The reverse transcription was performed according to the manufacturer's instruction. Resulting cDNA was precipitated overnight with 0.1 vol. 2 M NaCl, 2.5 vol. 100% ethanol, and 0.7 μl glycogen at −20°C, followed by 40 min centrifugation at 13,000 rpm and 4°C. After washing with 70% ethanol, the cDNA pellet was eluted in 25 μl DNase- and RNase-free water. cDNA was stored at −20°C or immediately used for the quantification of bacterial 16S rRNA.

### Quantitative PCR (qPCR) of bacterial 16S rRNA genes and transcripts

All qPCRs were performed with a Rotor-Gene Q cycler (Qiagen, The Netherlands) whereas each template DNA or cDNA was quantified in triplicates. The 20 μl reaction mixture consisted of 1-fold SensiFAST™ SYBR® No-ROX Kit (Bioline GmbH, The Netherlands), BSA (0.5 μg μl^−1^), 375–500 nM forward and reverse primers (Table S1), and 5 μl of diluted template DNA or cDNA of 2–6 ng μl^−1^. Negative controls consisting of DNase- and RNase-free water instead of template DNA were included in every qPCR run. Conditions for the primer sets targeting 16S rRNA gene of *Burkholderia, Dyella*, and *Paenibacillus* (Table S1) were as follows: 5 min initial denaturation at 95°C, 37 cycles of denaturation for 30 s at 95°C, annealing for 30 s at 64°C, elongation for 30 s at 72°C, and fluorescence signal detection for 15 s at 82°C. The 37th PCR cycle was followed by a melting curve analysis from 64 to 95°C with increments of 1°C. QPCR conditions for the primer sets targeting 16S rRNA gene of *Janthinobacterium* and *Pseudomonas* were the following: 5 min at 95°C, ensued by 37 cycles of denaturation for 30 s at 95°C, annealing for 20 s at 62°C, elongation for 20 s at 72°C, fluorescence signal detection for 15 s at 82°C, and a melting curve analysis from 62°C to 95°C immediately after the last qPCR cycle. Agarose gel electrophoresis of qPCR products displayed single bands of expected size (Table S1). Gene copy numbers were calculated according to a standard curve which was set up by serially diluting M13uni/rev PCR products of a pGEM-T vector containing a 16S rRNA gene fragment of the respected target organism (Zaprasis et al., 2010).

### Statistical analysis

All experiments were performed in triplicates. The statistical analysis of processed GC-MS data consistent with detected mass features per sample was conducted with MetaboAnalyst 3.0 (http://www.metaboanalyst.ca/MetaboAnalyst, Xia et al., 2015). Prior One-way ANOVA and multivariate analysis (PCA), data were filtered (interquantile range), and normalized (log transformation and auto scaling).

The statistical analysis on qPCR data as well as of CFU counts of *Pseudomonas sp.* AD021 growing as monoculture in soil microcosms supplied with or without ARE was performed with R 3.1.1 (http://www.r-project.org/) using ANOVA followed by Tukey's HSD test (De Mendiburu, 2014). To obtain normality of errors, data were log-transformed. Significance of enhanced CO_2_ production by *Pseudomonas sp.* AD021 growing in soil supplied with ARE was assessed by student's *t*-test. Differences revealed by statistical tests were considered significant for *P* < 0.05.

## Results

### Bacterial growth and volatile production in rhizospheric soil microcosms containing single or multiple species

**Figure 1:**
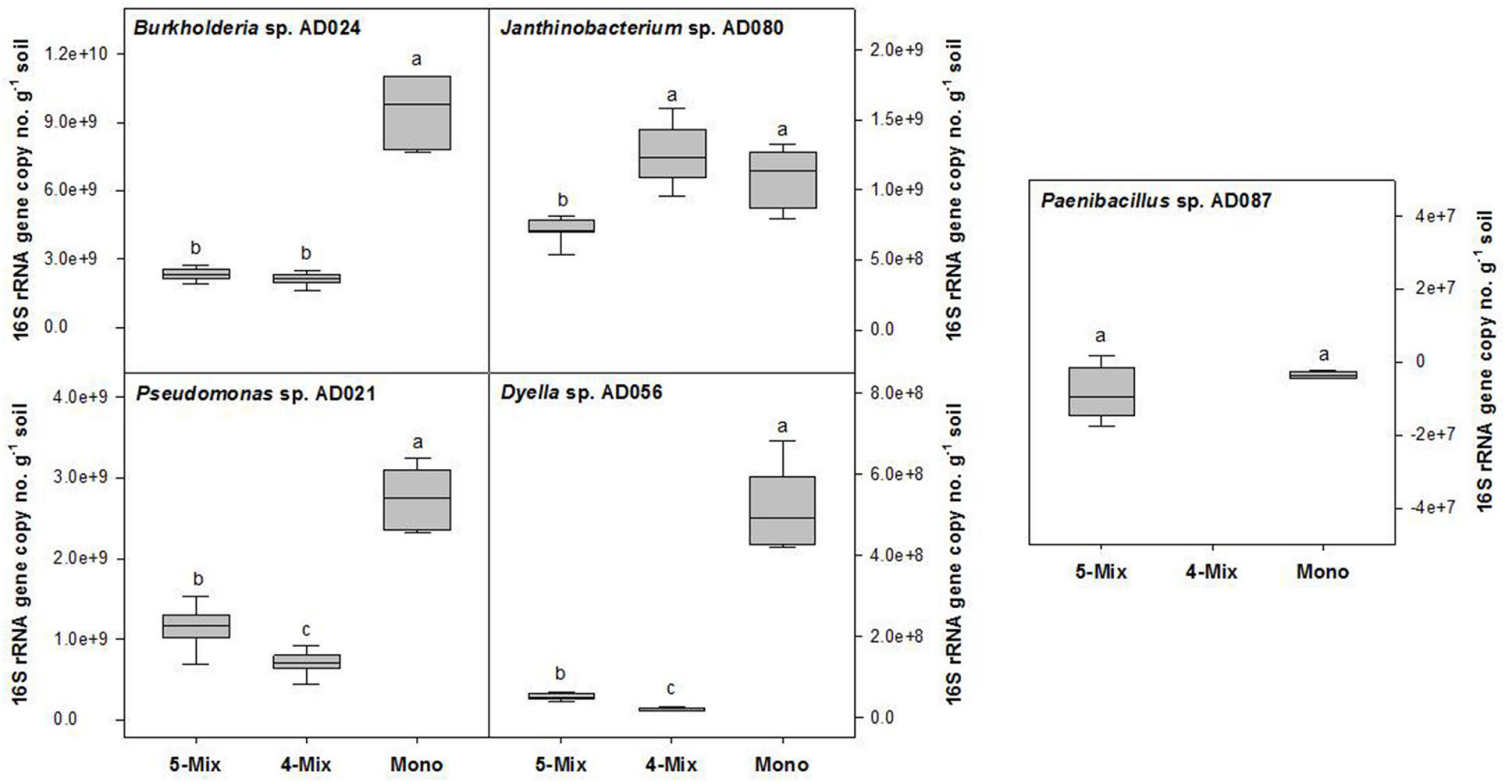
**Changes in bacterial 16S rRNA gene copy numbers per g soil after 4 days of incubation**. Bacteria were incubated in soil mixed with artificial root exudates as monoculture (Mono), bacterial mixture of five strains (5-Mix), or mixture of four strains excluding *Paenibacillus sp.* AD087 (4-Mix). Data represent values corrected for the starting time point t0. Different letters indicate significant difference (*P* < 0.05) between values resulted from One-way ANOVA and Tukey's HSD test.

For the rhizospheric soil microcosms, a sandy soil was selected with a low *in situ* availability of carbon. Metabolic activity and growth of bacteria were restricted without the addition of ARE to this soil (Figure S1). In the presence of the supplied carbon source, the growth and CO_2_ production of different rhizobacterial strains was assessed (Figure S2). Finally, five phylogenetic different bacterial strains (*Burkholderia sp*. AD024, *Dyella sp*. AD056, *Janthinobacterium sp*. AD080, *Pseudomonas sp*. AD021, and *Paenibacillus sp*. AD087) were selected. Those strains, except for *Paenibacillus sp*. AD087, showed a similar CO_2_ production profile. A maximal CO_2_production was reached after 6 days of incubation (Figure S2) which suggested that the supplied ARE as sole carbon source were fully consumed. Furthermore, the selected Gram-negative strains multiplied to a maximum of about 10^8^ CFU per g soil in the microcosms supplied with ARE (Figure S2). A quantification of 16S rRNA gene copy numbers revealed that the growth of *Burkholderia sp*. AD024, *Dyella sp*. AD056, and *Pseudomonas sp*. AD021 was significantly reduced in incubations with other bacteria in the ARE supplied soil (Figure 1). The Gram-positive strain *Paenibacillus sp*. AD087 grew poorly in the rhizospheric soil microcosms, both in monoculture and mixture (Figure 1 and Figure S2). However, in presence of *Paenibacillus sp*. AD087 the growth of the other bacteria in the mixture was affected. For instance, the 16S rRNA gene copy number (no.) of *Dyella sp.* AD056 and *Pseudomonas sp.* AD021 was significantly higher and for *Janthinobacterium sp.* AD080 significantly lower as compared to incubations without *Paenibacillus sp*. AD087 (Figure 1).

**Figure 2:**
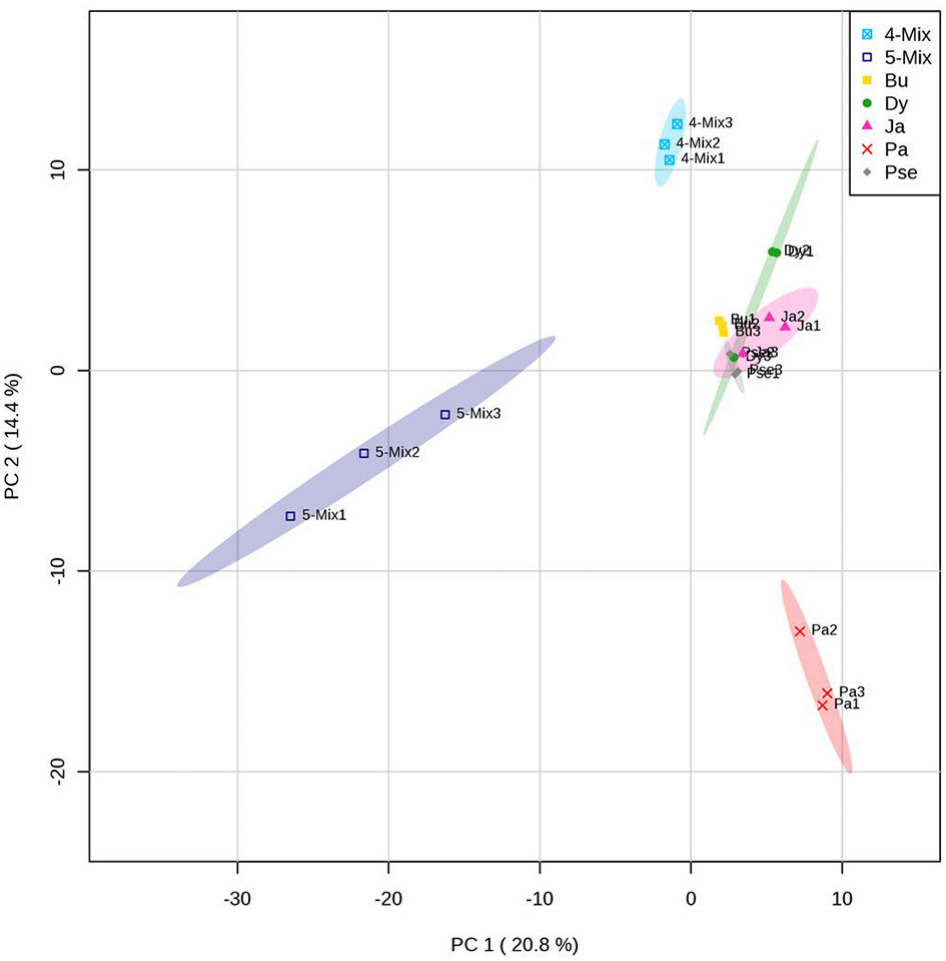
**PCA score plot of volatiles produced by bacteria in soil after 4 days of incubation in soil with artificial root exudates (ARE)**. The bacterial strains *Burkholderia sp.* AD024 (Bu), *Dyella sp.* AD056 (Dy), *Janthinobacterium sp*. AD080 (Ja), *Pseudomonas sp*. AD021 (Pse), and *Paenibacillus sp*. AD087 (Pa) were incubated as monoculture, mixture of five strains (5-Mix), or mixture of four strains excluding *Paenibacillus sp*. AD087 (4-Mix). Data represent multivariate analysis of mass features of volatiles only detected for microcosms containing bacteria.

A different blend of VOCs was produced by the bacterial mixture in comparison to the monocultures (Figure 2). The VOCs profile was different when the bacterial mixture consisted of five or four strains without *Paenibacillus sp*. AD087 (Figure 2). Volatiles released by the bacterial mixture consisted of alcohols, ketones, and esters as well as aromatic and organosulfur compounds. Some of those were also produced by the monocultures (Table 2). Most VOCs only released by the mixture of four bacterial strains excluding *Paenibacillus sp*. AD087 were alcohols and organic acids whereas VOCs released by for the mixture of all five strains consisted mainly of ketones, ester, and aromatic compounds. However, also numerous unknown volatile compounds were detected, especially for the bacterial mixture consisting of five strains (Table 2).

### Effect of microbial volatiles on starving bacteria

**Figure 3:**
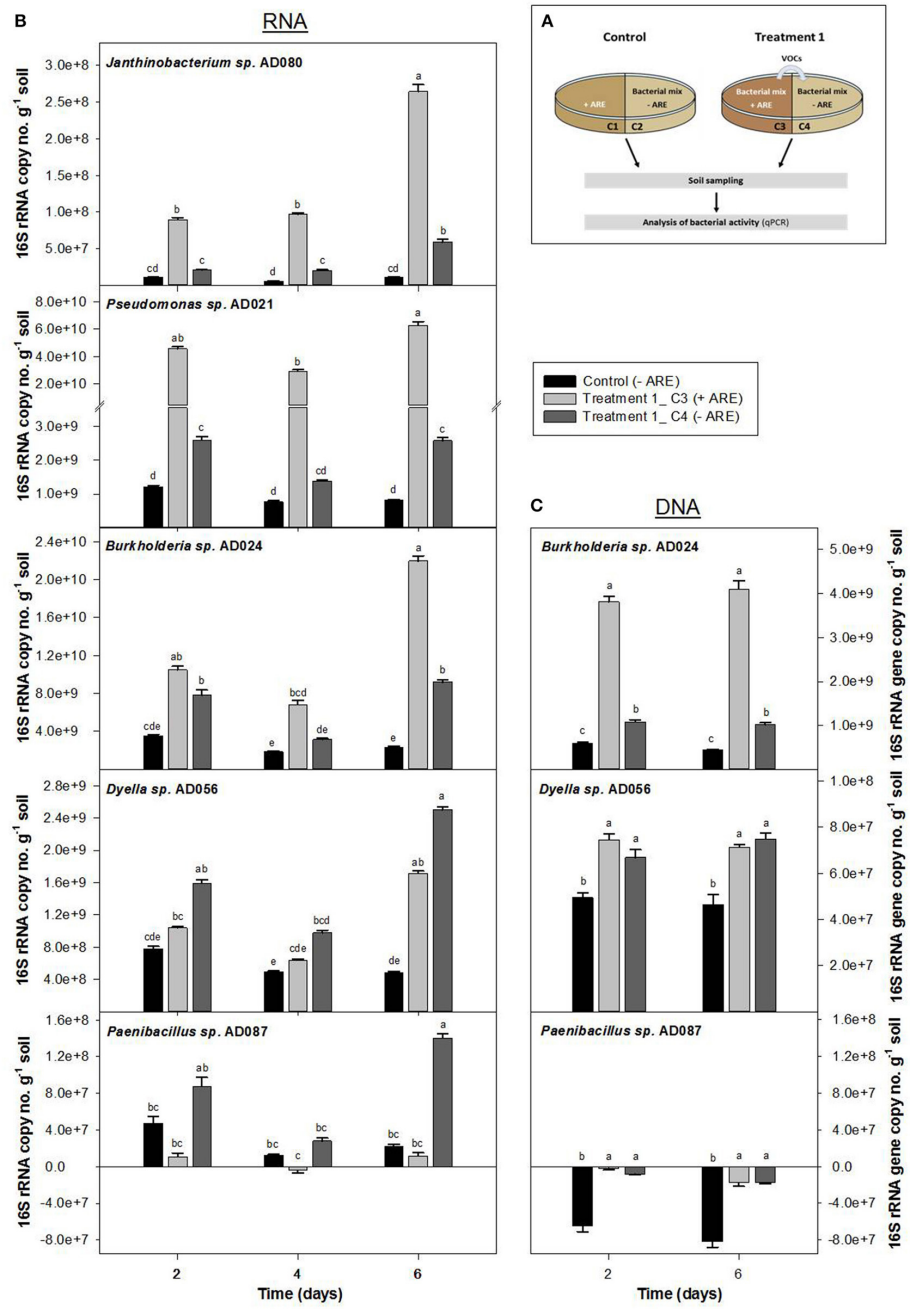
**Influence of bacterial volatiles on bacteria in nutrient-depleted soil**. Increase in 16S rRNA copy number **(B)** and 16S rRNA gene copy number per g soil **(C)** were determined for a bacterial community consisting of *Burkholderia sp.* AD024, *Dyella sp.* AD056, *Janthinobacterium sp.* AD080, *Pseudomonas sp.* AD021, and *Paenibacillus sp.* AD087 which was incubated in nutrient-poor soil. **(A)** In Treatment 1, bacteria in nutrient-depleted soil in compartment C4 (dark gray) were exposed to volatiles produced by bacteria supplied with artificial root exudates (ARE) in compartment C3 (light gray). The control compartment C2 (black) represents bacteria in nutrient-depleted soil not exposed to bacterial volatiles. Data represent mean (*n* = 6) and standard error corrected for the starting time point t0. Different letters indicate significant differences (*P* < 0.05) between values resulted from One-way ANOVA and Tukey's HSD test.

To test if volatiles produced during microbial interactions in a rhizosphere environment can stimulate starving microbes in the nutrient-depleted surrounding soil, soil microcosm experiments in two-compartment Petri-dishes with a mixture of all five bacterial strains were performed. Bacteria without additional carbon-source (compartment C4 of Treatment 1 and compartment C6 of Treatment 2) were exposed to volatiles produced by bacteria supplied with ARE (compartment C3 and C5, respectively) (Figure 3A and Figure S3A).

The 16S rRNA copy no. per g soil increased over time for bacteria supplied with ARE as well as for bacteria in the nutrient-depleted soil that were exposed to microbial VOCs (Figure 3B and Figure S3B). For the control microcosms, in which the ARE supplied compartment did not contain bacteria (Figure 3A and Figure S3A), the bacterial 16S rRNA copy no. per g soil did not significantly change over time. After 6 days of incubation, the 16S rRNA copy no. per g soil was significantly higher for all five bacterial strains in the nutrient-depleted soil exposed to microbial VOCs as compared to the control without exposure to microbial VOCs (Figure 3B). In case of *Dyella sp.* AD056 and *Paenibacillus sp.* AD087, the increase in 16S rRNA copy no. per g soil after 6 days of incubation was 1.5 and 12 times higher, respectively, when starving bacteria were exposed to microbial VOCs (compartment C4) as compared to bacteria growing in the ARE containing compartment (compartment C3) (Figure 3B). A similar trend was observed when bacteria were pre-incubated for 2 days in the soil before exposure to microbial VOCs (Figure S3B). Hence, volatiles released by mixtures of ARE-consuming bacteria significantly affected the activity of starving bacteria without ARE, based on an increase in 16S rRNA.

The 16S rRNA gene copy no. per g soil was measured for *Burkholderia sp.* AD024, *Dyella sp.* AD056, and *Paenibacillus sp*. AD087 to determine potential growth stimulation by exposure to bacterial VOCs under starving conditions. In case of *Burkholderia sp.* AD024 and *Dyella sp.* AD056, a significantly higher increase in 16S rRNA gene copy no. per g soil was observed for starving bacteria exposed to bacterial VOCs in comparison to the control (Figure 3C). In contrast, the 16S rRNA gene copy no. per g soil decreased over time for the strain *Paenibacillus sp.* AD087, even when nutrients were available or bacteria were exposed to VOCs (Figure 3C). Thus, the increase in activity by exposure to bacterial VOCs is coinciding with growth for the strains *Burkholderia sp.* AD024 and *Dyella sp.* AD056 but not for the Gram-positive *Paenibacillus sp.* AD087.

## Discussion

Most of the current knowledge about the possible functioning of microbial volatiles is based on *in vitro* studies under nutrient rich conditions (e.g., Kai et al., 2007; Vespermann et al., 2007; Zou et al., 2007). This is different from the nutrient-limiting conditions with which microbes have to deal in most soil environments. Here, we developed a soil model system mimicking more closely the natural situation occurring in and around the rhizosphere to reveal new insights into the ecological role of volatiles in microbial interactions in soil.

For bacteria incubated in rhizospheric soil microcosms, it was observed that the volatiles produced by the mixture differed from those produced by each strain. Some volatiles were only emitted by the bacterial mixture and not by the monocultures. The shift in the volatile blend can be due to competitive interactions (Garbeva et al., 2014a). This was indicated by a significant decrease in 16S rRNA gene copy number of *Burkholderia sp*., *Dyella sp*., and *Pseudomonas sp*. when they were growing in mixture with other bacterial strains. Besides inter-specific competitive interactions it was also observed that shifts in the bacterial community composition, i.e., bacterial mixture consistent of four or five different strains, influenced the volatile production. Therefore, as proposed by Mc Neal and Herbert (2009) changes in the VOCs profile can be a potential indicator of microbial community composition shifts. Indeed, recent studies pointed at a relationship between the composition of soil bacterial communities and that of VOCs (Hol et al., 2015; Van Agtmaal et al., 2015). Interestingly, the volatile emission by the bacterial mixture was strongly affected by the presence of the poorly-growing, non-abundant *Paenibacillus sp.* This is in line with recent studies revealing that low abundant microbes can play an important role in ecosystem functioning (Hol et al., 2010, 2015; Lynch and Neufeld, 2015). Furthermore, we observed that in the presence of the slow-growing *Paenibacillus sp.*, the 16S rRNA gene copy number of *Dyella sp*. and *Pseudomonas sp*. was significantly higher. Hence, non-abundant species can promote the growth of dominant species. On the other hand, they may also trigger the production of various growth-suppressing secondary metabolites such as antimicrobial volatiles (Jousset et al., 2014; Hol et al., 2015). In the presence of *Paenibacillus sp.*, several additional volatiles were released by the bacterial mixture which might be involved in the growth suppression of *Janthinobacterium sp.* besides non-vol atile antimicrobial compounds released by the interacting bacteria. Among those volatiles 2,5-bis(1-methylethy)-pyrazine was produced. It was recently reported that *Paenibacillus* can produce pyrazines with broad spectrum antimicrobial activity against bacteria, fungi and yeast (Rybakova et al., 2015). Here, the pyrazine-derivate was only detected for the bacterial mixture but not for *Paenibacillus sp.* in monoculture. However, it is possible that due to competitive interactions in the mixture the Gram-positive strain became active and started to produce the pyrazine compound.

Microorganisms are thought to use VOCs to influence other organisms living at a distance in the same soil environment (Stahl and Parkin, 1996). In the present study, we tested if volatiles produced by bacterial mixtures growing on root exudates can affect the activity of starving bacteria unable to access those root exudates. While microbes in the rhizosphere benefit from a constant flow of organic substrates (Effmert et al., 2012) volatile compounds released from the rhizosphere can represent an important carbon source to microorganisms in the nutrient-poor surrounding soil (Owen et al., 2007; Gramss and Bergmann, 2008; Ramirez et al., 2009). Besides, it has been suggested that volatiles produced by rhizosphere microorganisms could act as chemoattractants to the nutrient-rich environment around the roots (Garbeva et al., 2014a). Our results revealed that after 2 days of incubation, the activity of the starving bacteria *Burkholderia sp., Dyella sp.*, and *Pseudomonas sp.* was already stimulated by exposure to microbial VOCs. After 6 days of incubation, the activity of all five bacterial strains in the nutrient-depleted soil was significant increased by the exposure to microbial VOCs. Thus, volatiles released by rhizosphere-inhabiting bacterial communities can stimulate the activity of the surrounding starving bacteria in nutrient-depleted soil. The mechanism behind the activation of bacteria by volatiles, however, remains unclear. It was reported that volatiles can function as growth-promoting agents (Wheatley, 2002; Horii and Ishii, 2006; Garbeva et al., 2014a) which might explain the increase in bacterial activity by exposure to microbial VOCs. Quantification of 16S rRNA gene copy number revealed that the growth of the starving *Burkholderia sp.* and *Dyella sp.* was induced by exposure to microbial VOCs. Hence, the growth of starving bacteria in soil can be promoted by volatiles released from the rhizosphere. Bacteria are able to detoxify VOCs and/or use them as carbon and energy source. For instance, it was shown that *Pseudomonas fluorescens* is able to degrade the volatile compound alpha-pinene and to use it as a sole carbon source (Best et al., 1987; Kleinheinz et al., 1999). Furthermore, *Burkholderia* was reported as a toluene-degrader (Chen et al., 2015) and *Dyella* was abundant on a biofilter exposed to terpenes (Moe et al., 2013). In the current study, various terpenes and butylated hydroxytoluene were released by the bacterial mixture supplied with ARE in soil. It can be assumed that some of these volatiles stimulated the growth of *Burkholderia sp.* and *Dyella sp.* in the nutrient-depleted soil. This, however, needs to be verified in future studies.

Interestingly, while the activity of *Paenibacillus sp*. was strongly induced by exposure to microbial VOCs the growth was suppressed. As previously reported some VOCs can act as growth-inhibiting, toxic agents (Peñuelas et al., 2014) and induce stress response (Kim et al., 2013; Garbeva et al., 2014a). This might explain the strong induction in the activity and at the same time growth-suppression of *Paenibacillus sp*. by exposure to microbial VOCs. Another possible scenario for the strong increase in activity of *Paenibacillus sp*. is the induction of motility to escape from the toxic environment. A stimulation of motility by exposure to microbial VOCs was already reported for several bacterial species (Kim et al., 2013; Hagai et al., 2014; Garbeva et al., 2014a). For microbes living in the heterogeneous soil environment, an activation of motility by volatiles may be important to move toward nutrient-rich regions or to escape from hostile areas. Hence, volatiles in soil can provide important information on the quality of the nearby surroundings.

Besides the role as growth-promoting or growth-suppressing agents (Peñuelas et al., 2014), volatiles can also induce the production of non-volatile secondary metabolites. For instance, it was recently reported that microbial volatiles induce the production of inhibiting secondary metabolites in *Pseudomonas fluorescence* against Gram-positive bacteria (Garbeva et al., 2014a). Thus, the growth suppression of *Paenibacillus sp*. may be also due to non-volatile secondary metabolites which production was activated by microbial volatiles.

In conclusion, this study revealed that the blend of released volatiles from the rhizosphere is affected by inter-specific competitive interactions and shifts in the microbial community composition. Moreover, the presence of non-abundant, slow-growing species can strongly influence the volatile production by other dominant species. Based on our results and other recent studies it is evident that microbial volatiles in soil can serve multiple roles as C-source, defense metabolites, chemoattractant, repellants or other unknown so far. Hence, volatiles released by rhizosphere-inhabiting microbial communities can have a significant long distance effect on starving microorganisms in the surrounding, nutrient-depleted soil.

### Conflict of interest statement

The authors declare that the research was conducted in the absence of any commercial or financial relationships that could be construed as a potential conflict of interest.
