# Supplementary material for: A fragrant neighborhood: volatile mediated bacterial interactions in soil
Source: Front Microbiol. 2015 Nov 3;6:1212. doi: 10.3389/fmicb.2015.01212 (PMC4631045; doi:10.3389/fmicb.2015.01212)
Supplement: Supplementary file 1 [file Table1.PDF]

Table S1. Primers and qPCR conditions used in this study.

Primers[1]	Sequence (5'→3')	Size (bp)	Primer conc.[2] (nM)	Target	Reference
BKH812F	CCCTAAACGATGTCAACTAGTTG	402	500	*Burkholderia*	Bergmark et al., 2012
BKH1249R	ACCCTCTGTTCCGACCAT		500	*Burkholderia*	Bergmark et al., 2012
DY456F	CTAATATCCGGTGGGGCTGAC	267	400	*Dyella*	This study
DY722R	TTCGCCACTGATGTTCCTCC		400	*Dyella*	This study
PA1249F	GTACAACGGGCTGCGAAATC	198	400	*Paenibacillus*	This study
PA1446R	CCACCGACTTCGGGTGTTAT		400	*Paenibacillus*	This study
Pse853F	CGCATTAAGTTGACCGCCTG	174	375	*Pseudomonas*	This study
Pse1026R	ATGTTCCCGAAGGCACCAAT		375	*Pseudomonas*	This study
JA1223F	ATGGTACATACAGAGCGCCG	194	400	*Janthinobacterium*	This study
JA1416R	CTGGTAAAACCCGCTCCCAT		400	*Janthinobacterium*	This study

[1] Forward primer is indicated by F and reverse primer by R.
[2] Final primer concentrations in 20 µl qPCR reaction mix.
