# Supplementary material for: A fragrant neighborhood: volatile mediated bacterial interactions in soil
Source: Front Microbiol. 2015 Nov 3;6:1212. doi: 10.3389/fmicb.2015.01212 (PMC4631045; doi:10.3389/fmicb.2015.01212)
Supplement: Supplementary file 2 [file Image1.PDF]

Figure S1

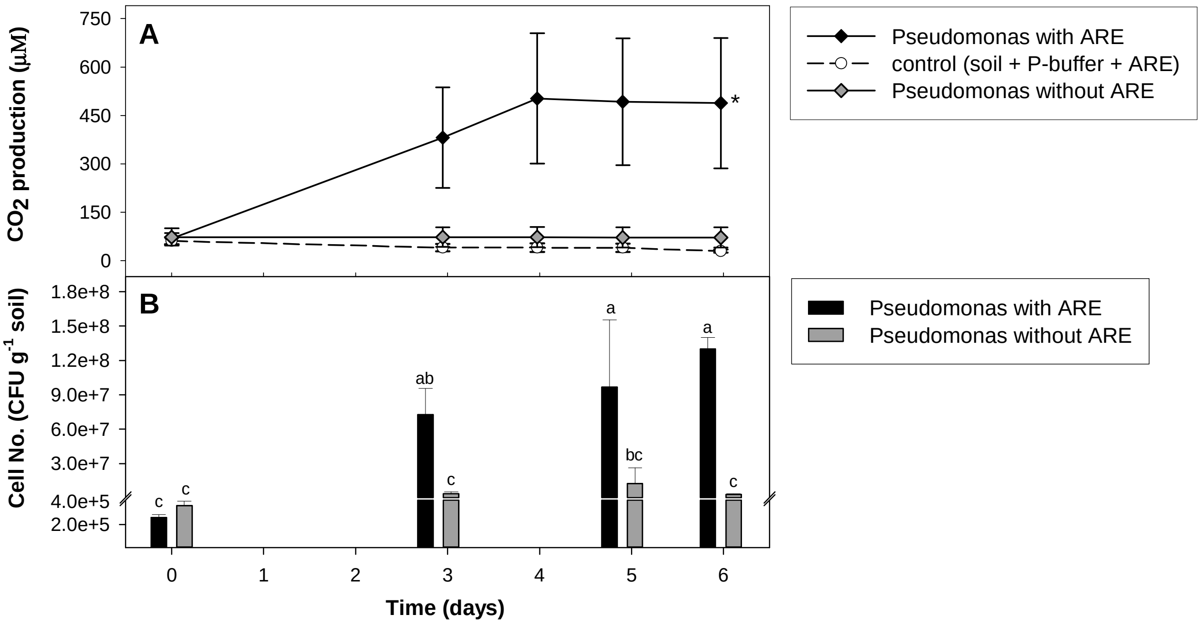

Figure S1 Evidence for nutrient-limited growth in soil microcosms. Cumulative CO_2-production (A) and growth (B) was determined for the rhizospheric strain *Pseudomonas sp.* AD021. Data represent mean (n = 3) and standard error. Star indicates a significant higher CO_2 production ($P<0.05$) for soil microcosms of *Pseudomonas* supplied with artificial root exudates (ARE). Different letters indicate significant difference ($P < 0.05$) between cell numbers (no.) resulted from one-way ANOVA and Tukey's HSD test.
