# Supplementary material for: A fragrant neighborhood: volatile mediated bacterial interactions in soil
Source: Front Microbiol. 2015 Nov 3;6:1212. doi: 10.3389/fmicb.2015.01212 (PMC4631045; doi:10.3389/fmicb.2015.01212)
Supplement: Supplementary file 3 [file Image2.PDF]

Figure S2

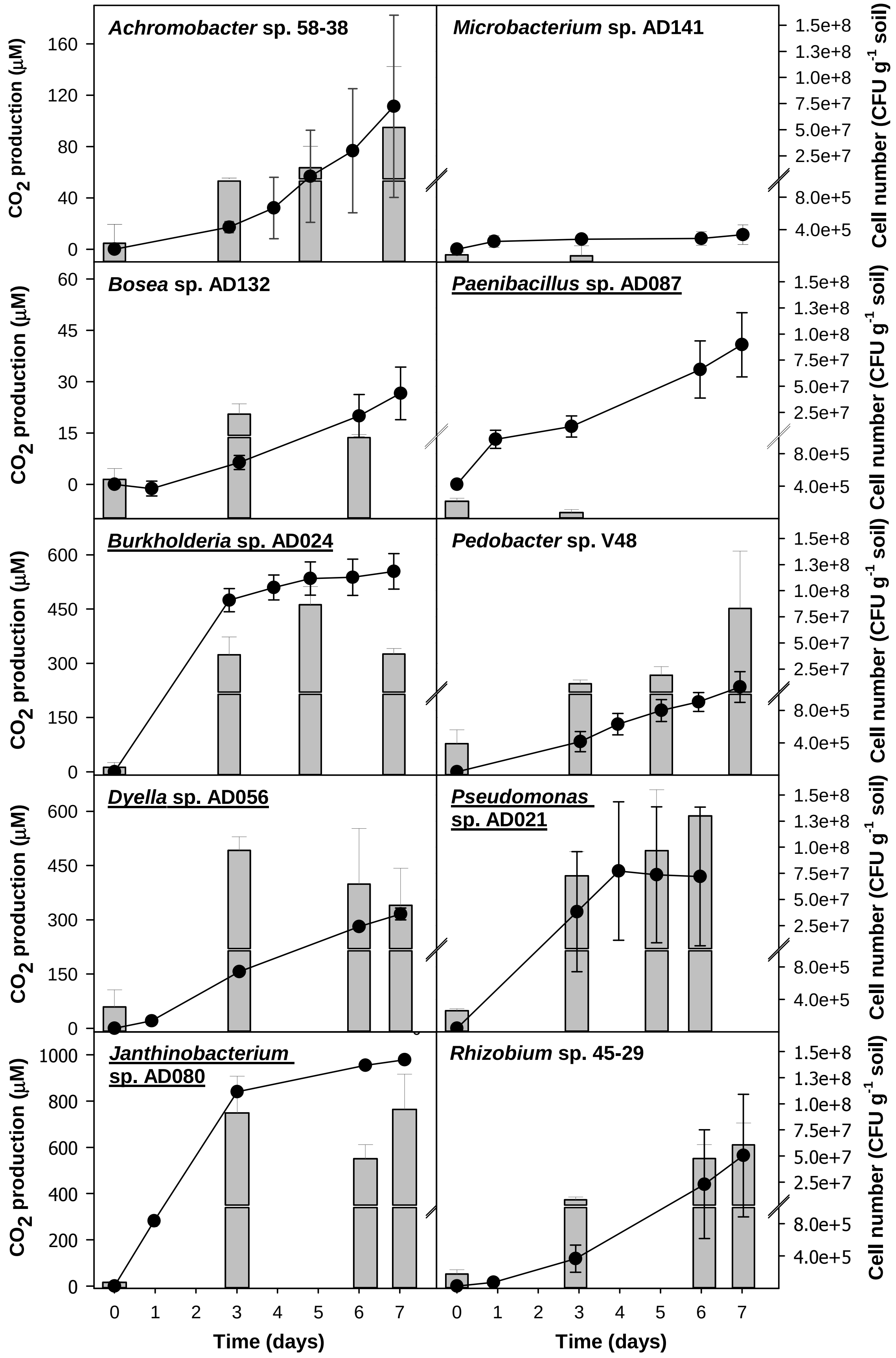

Figure S2 Growth (grey bars), represented by increase in Colony forming units (CFU) per g soil, and CO_2 production of bacteria (black dots) incubated in rhizospheric soil microcosms supplied with ARE. Data represent mean (n=3) and standard error. Bacterial strains selected for further analysis are underlined.
