# Supplementary material for: A fragrant neighborhood: volatile mediated bacterial interactions in soil
Source: Front Microbiol. 2015 Nov 3;6:1212. doi: 10.3389/fmicb.2015.01212 (PMC4631045; doi:10.3389/fmicb.2015.01212)
Supplement: Supplementary file 4 [file Image3.PDF]

Figure S3

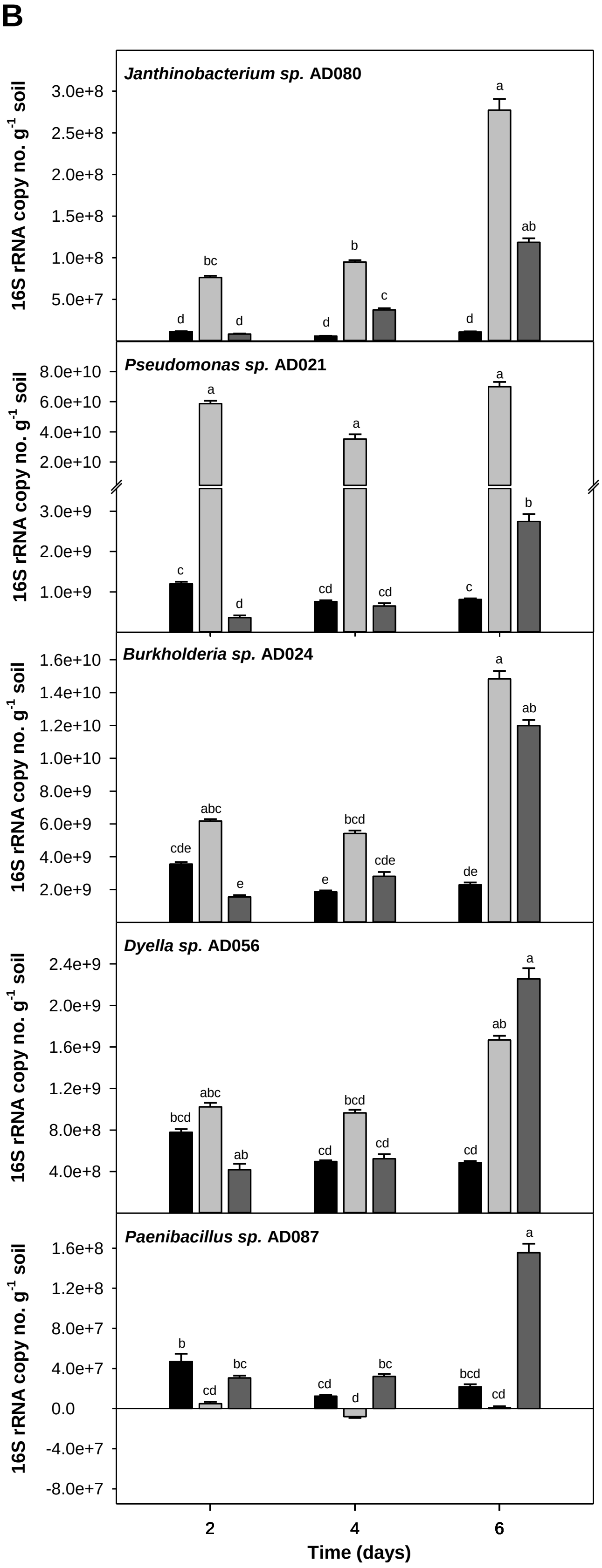

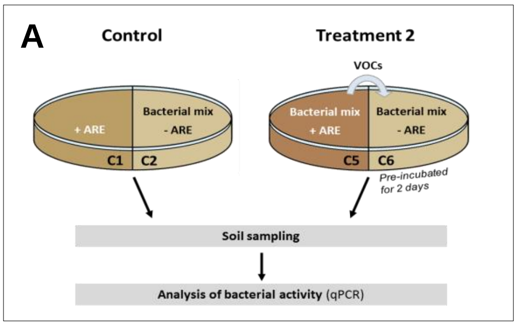

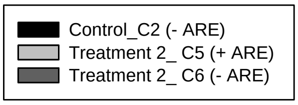

Figure S3 Influence of bacterial volatiles on nutrient-depleted bacteria. (B) 16S rRNA copy number per g soil were measured for a bacterial community of *Burkholderia sp*. AD024, *Dyella sp*. AD056, *Janthinobacterium sp*. AD080, *Pseudomonas sp*. AD021, and *Paenibacillus sp*. AD087 which was incubated in nutrient-poor soil. (A) In Treatment 2, bacteria in compartment C5 (dark grey) were pre-incubated for two days in nutrient-depleted soil before exposure to volatiles originating from bacteria supplied with artifi cial root exudates (ARE) in compartment C6 (light grey). The control compartment C2 (black) represents bacteria in nutrient-depleted soil not exposed to bacterial volatiles. Data represent mean (n = 9) and standard error corrected for the starting time point t0. Different letters indicate significant difference (P<0.05) between values resulted from one-way ANOVA and Tukey's HSD test.
